# Acute effects of hypouricemia on endothelium, oxidative stress, and arterial stiffness: A randomized, double‐blind, crossover study

**DOI:** 10.14814/phy2.15018

**Published:** 2021-08-26

**Authors:** Benjamin De Becker, Emeline Hupkens, Laurence Dewachter, Catherine Coremans, Cédric Delporte, Pierre van Antwerpen, Thierry Franck, Karim Zouaoui Boudjeltia, Pierre Cullus, Philippe van de Borne

**Affiliations:** ^1^ Department of Cardiology Erasme Hospital Université Libre de Bruxelles Brussels Belgium; ^2^ Laboratory of Physiology and Pharmacology Faculty of Medicine Université Libre de Bruxelles Brussels Belgium; ^3^ RD3 – Pharmacognosy, Bioanalysis and Drug Discovery & Analytical Platform of the Faculty of Pharmacy (APFP) Faculty of Pharmacy Université Libre de Bruxelles Brussels Belgium; ^4^ Centre of Oxygen, Research and Development Institute of Chemistry B 6a University of Liege ‐ Sart Tilman Liège Belgium; ^5^ Laboratory of Experimental Medicine (ULB 222) Medicine Faculty Université Libre de Bruxelles CHU de Charleroi, Hopital Vesale Montigny‐le‐Tilleul Belgium; ^6^ Biostatistics department, Medicine Faculty Université Libre de Bruxelles Brussels Belgium

**Keywords:** febuxostat, NO synthase, rasburicase, renin–angiotensin–aldosterone system

## Abstract

We hypothesized acute moderate and drastic reductions in uric acid concentration exert different effects on arterial function in healthy normotensive and hypertensive adults. Thirty‐six adults (aged 58 [55;63] years) with or without primary hypertension participated in a three‐way, randomized, double‐blind, crossover study in which [placebo] and [febuxostat] and [febuxostat and rasburicase] were administered. Febuxostat and rasburicase reduce the uric acid concentration by xanthine oxidoreductase inhibition and uric acid degradation into allantoin, respectively. Endothelial function was assessed in response to acetylcholine, sodium nitroprusside, heating (with and without nitric oxide synthase inhibition) using a laser Doppler imager. Arterial stiffness was determined by applanation tonometry, together with blood pressure, renin–angiotensin system activity, oxidative stress, and inflammation. Uric acid concentration was 5.1 [4.1;5.9], 1.9 [1.2;2.2] and 0.2 [0.2;0.3] mg/dL with [placebo], [febuxostat] and [febuxostat–rasburicase] treatments, respectively (*p* < 0.0001). Febuxostat improved endothelial response to heat particularly when nitric oxide synthase was inhibited (*p* < 0.05) and reduced diastolic and mean arterial pressure (*p* = 0.008 and 0.02, respectively). The augmentation index decreased with febuxostat (ANOVA *p* < 0.04). Myeloperoxidase activity profoundly decreased with febuxostat combined with rasburicase (*p* < 0.0001). When uric acid dropped, plasmatic antioxidant capacity markedly decreased, while superoxide dismutase activity increased (*p* < 0.0001). Other inflammatory and oxidant markers did not differ. Acute moderate hypouricemia encompasses minor improvements in endothelial function, blood pressure, and arterial stiffness.

**Clinical Trial Registration**: NCT03395977, https://clinicaltrials.gov/ct2/show/NCT03395977

## Graphical abstract

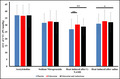

**What is the central question of this study?**

Cardiovascular effects of acute reduction in uric acid level are still not well understood. By the means of two pharmacological agents, we compared the effects of acute moderate and severe reduction in uric acid on arterial function in adults with or without primary hypertension.

**What are the main finding and its importance?**

Acute moderate hypouricemia slightly improved endothelial function and modestly reduced blood pressure and arterial stiffness. Hypouricemia is associated with reduced plasma antioxidant capacity. Myeloperoxidase activity, a potential marker of cellular oxidative stress, is strongly reduced after extreme diminution in uric acid concentration.

## 1 INTRODUCTION

Uric acid (UA) is a plasma antioxidant scavenging oxidants such as hydroxyl radicals and superoxide anion (Johnson et al., 2013). In addition, it plays a pro‐oxidant and proinflammatory role inside cells (Mazzali et al., 2010). Many epidemiological studies have associated UA with cardiovascular disease especially hypertension (Johnson et al., 2005), considering UA as an independent cardiovascular risk factor (Borghi et al., 2018). The risk of hypertension increases by 13% for each mg/dL increase in UA concentration (Grayson et al., 2011). UA exerts deleterious effects on endothelial cells and the renin–angiotensin system (RAS) in kidneys. Indeed, UA produces a UA dependent‐hypertension by endothelial dysfunction (reduction in nitric oxide [NO] bioavailability) and RAS activation. Then, UA is responsible for renal arteriopathy leading to salt‐sensitive hypertension (De Becker, Borghi, et al., 2019). Additionally, UA is associated with endothelial dysfunction, arterial stiffness, oxidative stress, and inflammation (Kanbay et al., 2013).

UA is the final product of purine metabolism and metabolized by xanthine oxidase (XO) which is one of the major reactive oxygen species (ROS) producers, playing a role in endothelial function (De Becker, Borghi, et al., 2019) Inhibition of XO by allopurinol has shown many benefits in terms of improving endothelial function (Alem, 2018; Cicero et al., 2017; Higgins et al., 2011; Kanbay et al., 2014), decreasing blood pressure (BP) (Agarwal et al., 2013; Qu et al., 2016), ameliorating arterial stiffness (Deng et al., 2016) and reducing inflammation and oxidative stress (Higgins et al., 2011; Yiginer et al., 2008); however, no recommendations have been yet established (Gois and Erdm, 2017). Interventional studies on acute effects of UA reduction remain rare and exceptionally include febuxostat (a specific XO inhibitor) or rasburicase (a recombinant enzyme that degrades UA into allantoin). It remains unclear whether UA plays a deleterious role in the cardiovascular system and whether its acute reduction could be beneficial. Moreover, extreme hypouricemia appears to be potentially harmful and is associated with cardiovascular events (Perez‐Gomez et al., 2019). Indeed, in patients with hypertension, lower UA levels are also associated with more cardiovascular events (J‐shape relationship) (Verdecchia et al., 2000).

Therefore, we designed a study to understand the acute effects of moderate and severe reduction in UA levels. Our first study on normouricemic young males showed that extreme UA reduction altered heat‐induced endothelium‐dependent vasodilation, slightly reduced systolic blood pressure, and markedly reduced myeloperoxidase (MPO) activity (De Becker, Coremans, et al., 2019). We hypothesized in this second work on middle‐aged adults that an acute moderate decrease in UA could be beneficial for the cardiovascular system in contrast to a drastic reduction.

## 2 METHODS

The data supporting the findings of this study are available from the corresponding author upon request.

### 2.1 Ethics approval

The protocol was reviewed and approved by the ethics committee of the Erasme Hospital (reference P2017/296; NCT03395977). The study was conducted in accordance with the principles outlined in the Declaration of Helsinki. Written informed consent was obtained from all participants.

### 2.2 Population and design

Between May and December 2019, 36 participants aged between 40 and 66 years of both genders with or without primary hypertension were recruited at the Erasme Hospital (Brussels, Belgium). All participants were white individuals and had a normal physical examination. Active smokers and excessive alcohol consumers (more than three units per day) were excluded. Primary hypertensive participants under therapy were required to have RAS blockers to respect homogeneity. Treatment was required to be unchanged for at least 6 months before inclusion. Patients with glucose‐6‐phosphate dehydrogenase (G6PD) deficiency were excluded. At the time of inclusion, BP was measured after at least 5 min of seated rest in a quiet room (automatic BP monitor Omron 705 IT, Kyoto, Japan). Three measurements were taken with an interval of 1−2 min. If BP measurements differed by >10 mmHg, further measurements were taken. Hypertensive participants consisted of participants with a BP above 140/90 mmHg and participants treated for hypertension.

**FIGURE 1:**
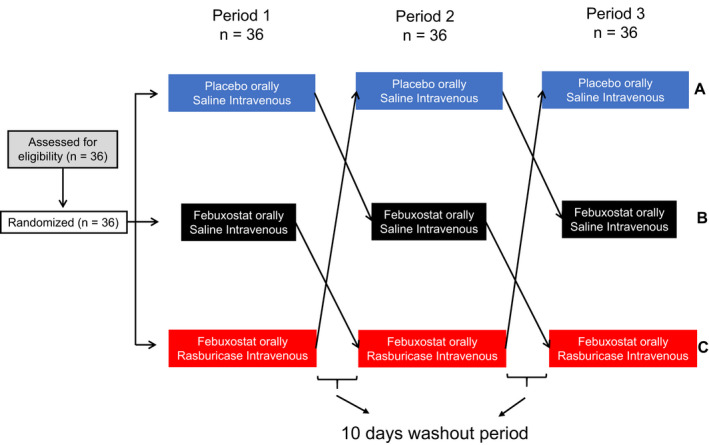
Flowchart diagram. Thirty‐six adult volunteers were enrolled and randomized into three groups (ABC, BCA, or CAB). Each group comprised three treatments separated by a 10‐day washout period. Treatments were placebo and intravenous (IV) saline (A), febuxostat administered orally and IV saline (B), and febuxostat administered orally and IV rasburicase (C)

Participants were enrolled in a randomized, double‐blind, placebo‐controlled, three‐way (A, B, C), crossover study with a 10‐day washout period between experimental sessions. Each participant completed three sessions of 4 days. Pills were given on days 1 to 3 (lactose as placebo or 240 mg febuxostat/day). On day 3, participants were infused with saline or 3 mg rasburicase reconstituted in 50 ml of saline. Experimental measures took place on day 4. Pills (placebo or febuxostat packaged in identical white capsules) and infusions were prepared by a pharmacist in accordance with the randomization protocol (three treatment orders were established: ABC, BCA, or CAB). Treatment [A] was placebo orally administered and intravenous (IV) saline; [B] was febuxostat orally administered and IV saline; and [C] was febuxostat orally administered and IV rasburicase (Figure 1) similarly to our previous work (De Becker, Coremans, et al., 2019). The half‐lives of febuxostat and rasburicase are, respectively, 1.3 to 15.8 h (Khosravan et al., 2006) and 16 to 21 h (Ueng, 2005). The order of the treatments was determined prior to inclusion and allocated to participants following enrolment. Volunteers and investigators were blinded to the medications administered during the study. The highest tested dosage of febuxostat was administered to ensure XO would be completely blocked (Kamel et al., 2016). A 24 h period between rasburicase or saline infusion and measurements was observed to achieve the lowest concentration of UA, as previously described (Waring et al., 2007). All investigations (biological samples, microvascular function, hemodynamic parameters, and arterial stiffness) were performed on day 4 in a quiet room maintained at 22 ± 2℃. Measurements were performed in the morning at identical time points for all participants. Furthermore, participants refrained from consuming alcohol, caffeine‐based drinks, intense exercise, and highly salty, fatty, or sweet meal at least 48 h before endothelial function assessment. Participants fastened for 12 h on recruitment day and days 3 and 4 of each session.

A blood sample was collected at recruitment and on day 4 of each session, whereas the urine sample was collected on day 4. Venous samples for UA measurement were transported on ice to inhibit potential rasburicase activity. Standard biological analyses were performed in our hospital directly after collection. The remaining serum, plasma (following centrifugation at 3500 g for 15 min at 20℃), and urine samples were isolated and stored at −80℃ in our biobank (BE_BERA1; Biobanque Hôpital Erasme‐ULB (BERA); BE_NBWB1; Biothèque Wallonie Bruxelles (BWB); BBMRI‐ERIC) until analysis.

### 2.3 Primary outcome

#### 2.3.1 Endothelial function

The microcirculatory vasomotor function was assessed by a laser Doppler Imager (Moor Instrument, Axminster, UK) that measured blood flow in a skin surface area of 3.8 cm² (De Becker, Coremans, et al., 2019; Wauters et al., 2013). Acetylcholine (ACh), sodium nitroprusside (SNP), L‐N^G^‐Nitro arginine methyl ester (L‐NAME, a NO synthase inhibitor), and saline solution were used for iontophoresis as previously described (Dreyfuss et al., 2013; Wauters et al., 2013). ACh‐ and SNP‐induced hyperemia were applied to assess the endothelium‐dependent and endothelium‐independent vasomotor response, respectively. To assess the endothelial vasomotor response, heat‐induced hyperemia after L‐NAME and saline iontophoresis were also studied. L‐NAME facilitated the analysis of the NO‐independent but endothelial‐dependent vasodilation response to heat. This non‐invasive technique is central for the assessment of peripheral endothelial function (Kubli et al., 2000). The measurements were performed in a quiet room, in the supine position under carefully standardized conditions.

Drug iontophoresis was continued for 22.5 min in order to achieve maximal skin vasodilation. ACh and SNP solutions were prepared to obtain a final concentration of 2 g/100 ml in deionized water; 2.5 ml of these solutions were introduced into the anode (ACh electrode) and the cathode (SNP electrode) chambers on the right forearm. L‐N^G^‐Nitro arginine methyl ester (L‐NAME) was diluted in sterile water to obtain a 20 mM solution; 2.5 ml of this solution and saline were introduced into the anode (L‐NAME electrode) and the cathode (saline electrode) chambers on the left forearm. Electric current was generated by an iontophoresis controller (MIC 2, Moor Instruments Ltd, Axminster, United Kingdom), which was set to apply a current of 100 μA. On the left forearm, the skin was heated to 44℃ using dedicated skin heater electrodes and a temperature monitor (SH02, Moor Instruments Ltd, Axminster, UK) after L‐NAME and saline iontophoresis (Wauters et al., 2013).

Twelve scans were obtained from the laser Doppler imager, with the first two scans corresponding to the baseline cutaneous flow. Thirty minutes prior to iontophoresis, 5% Emla^®^ cream (lidocaine 2.5% and prilocaine 2.5%; AstraZeneca, London, UK) was applied to the anterior face of both forearms to prevent nonspecific vasodilatation induced by the electric current.

The skin response to ACh and SNP is monophasic. The heat response is biphasic and depends on the endothelial system, adrenergic nerves, and sensory nerves (Johnson & Kellogg, 2010). The first phase is non‐specific and primarily mediated by transient axon reflex vasodilatation and marginally by NO. The second (plateau) phase is mainly related to NO release by the endothelium (Johnson & Kellogg, 2010). Therefore, we only compared the late‐phase response (5th to 10th scan) between sessions in terms of the skin response to heat, as in our previous studies (Wauters et al., 2013). Data were expressed in cutaneous vascular conductance calculated (CVC) by normalizing the cutaneous blood flow in perfusion units (PU) with mean arterial pressure (MAP) in mmHg. To facilitate the comparison, we used the area under the curve (AUC) of CVC for all laser Doppler recordings.

### 2.4 Secondary outcomes

#### 2.4.1 Biological measures

The estimated glomerular filtration rate (eGFR) was calculated with the CKD‐EPI Creatinine Equation (Levey et al., 2009). Homocitrulline/lysine and 3‐chlorotyrosine/tyrosine ratios, allantoin, interleukin (IL)‐6 and IL‐8, MPO activity and concentration, and malondialdehyde (MDA) were used to assess inflammation and oxidative stress. 3‐chlorotyrosine is a specific oxidation product of tyrosine by MPO, and MPO may catalyze the formation of homocitrulline from lysine. These products were measured by acid hydrolysis, derivatization, and liquid chromatography‐mass spectrometry tandem (LC‐MS/MS) (Delporte et al., 2012). MDA is an end‐product formed through the degradation of specific lipid peroxidation products. Allantoin is a marker of oxygen‐free radical load in individuals not receiving rasburicase and is the result of a nonenzymatic reaction between UA and ROS. Allantoin was measured by LC‐MS‐MS. The activity of MPO, a major oxidative enzyme, was measured using the SIEFED method (Franck et al., 2015). Plasma antioxidant properties were assessed by a superoxide dismutase (SOD) assay, which determines the activity of all types of SOD and Ferric Antioxidant Status Detection Kit. Soluble InterCellular Adhesion Molecule 1 (sICAM‐1) and sP‐selectin are adhesion proteins involved in endothelial dysfunction. Endothelial analysis was completed by nitrites measurement. Laboratory RAS analyses were completed with the measurement of angiotensin II. Details about measurements and assays list can be found in the Supplementary Material.

#### 2.4.2 Hemodynamic parameters and arterial stiffness

BP and heart rate (HR) were measured using an automatic BP monitor (Omron 705 IT, Kyoto, Japan). The cuff was adapted to the arm size and measures were taken by a trained doctor. Pulse pressure and MAP were calculated from BP data. Muscular arteries and aortic stiffness were assessed by the pulse wave velocity (PWV) through the carotid‐radial and carotid‐femoral applanation tonometry, respectively (cr‐ and cf‐PWV) (Mitchell et al., 2010). Augmentation index and heart rate corrected augmentation index (AIx and AIx75, arterial wave reflection) were also used as arterial stiffness markers and measured via the radial artery applanation tonometry technique. PWV and AIx were assessed non‐invasively using a fully automated device (SphygmoCor; Atcor Medical, Sydney, NSW, Australia), as previously described (Adamopoulos et al., 2009).

#### 2.4.3 Adverse effects

Participants were advised that gastrointestinal and other less common adverse effects could potentially occur. All participants were requested to report any complaints to the principal investigator.

### 2.5 Data analysis and statistics

Variables were expressed as mean (standard deviation) when normally distributed, or otherwise expressed as median values [Quartile 1; Quartile 3]. Data analysis for the crossover design was performed using a global linear model for repeated measures (mixed ANOVA). The conditions (placebo, febuxostat, or febuxostat–rasburicase) were used as a *within*‐*subjects* factor and the sequence order of the sessions (ABC, BCA, or CAB) as a *between*‐*subjects* factor to assess the carryover effect (effect of a condition on another). Non‐normally distributed data were analyzed using the non‐parametric Friedman test for repeated measures or a paired Wilcoxon test when appropriate. Binary variables, such as adverse effects, were analyzed using a Chi‐square test. Bonferroni correction was applied for multiple comparisons. Correlation between variable and UA concentration was performed using the Spearman's correlation test. A *p* value of <0.05 was considered statistically significant. All statistical analyses were performed using SPSS version 22.0 (IBM Corporation, Armonk, New York, NY, USA). Subgroup analyses were performed according to sex and hypertension status. Independent‐samples T‐test and the non‐parametric Mann–Whitney test were used to compare variables within the subgroups.

Sample size calculation was performed through G3*Power to obtain a power of 80% and an error alpha of 5%. We estimated the size effect of the primary outcome from our previous study (De Becker, Coremans, et al., 2019). We needed at least 21 participants in each treatment group to get the desired power. For this endpoint, we can thus properly interpret the results including the subgroups analysis for men and normotensive participants (*n* = 23).

For secondary outcomes, the size effects were larger and the requested numbers of participants lower. Therefore, all subgroup analyses were allowed to be performed without any reserve.

## 3 RESULTS

### 3.1 Biological characteristics of the participants at baseline

Thirty‐six subjects were randomized for all three treatment sequences (Figure 1 and Table 1). UA concentration was 4.4 (1.8) in females and 6.0 (1.2) mg/dl in males (*p* < 0.05).

**TABLE 1:** Characteristics of the study participants at baseline (*n* = 36)

Clinical	
Age (years)^a^	58 [55; 63]
BMI (kg/m²)	25.6 (3.6)
Females, *n* (%)	13 (36.1)
SCORE Risk (%)^a^	1.0 [0.3; 2.0]
Hypertension, *n* (%)	13 (36.1)
Antihypertensive, *n* (%)	5 (13.9)
Biological	
Hb (g/dl)	14.5 (1.2)
G6PD (U/g of Hb)	9.6 (1.8)
CRP (mg/l)^a^	1.1 [0.5; 2.0]
eGFR (ml/min/1.73 m²)	87.4 (12.0)
Sodium (mmol/L)^a^	141.0 [140.0; 142.0]
Potassium (mmol/L)	4.1 (0.6)
Uric acid (mg/dl)	5.4 (1.2)
LDH (U/L)	178.9 (30.6)
Cholesterol (mg/dl)	196.3 (34.8)
Triglyceride (mg/dl)^a^	79.0 [64.3; 112.0]
HDL (mg/dl)	62.9 (14.4)
Cholesterol/HDL ratio^a^	3.3 [3.0; 4.1]
LDL (mg/dl)	114.5 (36.0)
non‐HDL (mg/dl)	133.4 (41.4)
ApoA1 (mg/dl)	160.6 (19.8)
ApoB (mg/dl)	110.2 (30.0)
ACE (U/L)	34.4 (14.4)
Glucose (mg/dl)^a^	96.0 [92.3; 98.8]

Abbreviations: BMI, body mass index; Hb, haemoglobin; G6PD, glucose‐6‐phosphate dehydrogenase; CRP, C‐reactive protein; eGFR, estimated glomerular filtration rate; LDH, lactate dehydrogenase; HDL, high‐density lipoproteins; LDL, low‐density lipoproteins; Apo, apolipoprotein; and ACE, angiotensin‐converting enzyme.

^a^ not normally distributed.

### 3.2 Biological analyses

The plasma and urinary concentration of UA decreased in participants treated with febuxostat and febuxostat–rasburicase (*p* < 0.0001). There was no significant difference observed between other biological variables. Urinary sodium‐ and protein–creatinine ratios were similar between treatments. There was no carryover effect (Table 2).

**TABLE 2:** Biological and hemodynamic parameters

Measures	Placebo (*n* = 36)	Febuxostat (*n* = 36)	Febuxostat and Rasburicase (*n* = 36)	*p*			
				*ANOVA*	*P* versus *FX*	*P* versus *FX*‐*R*	*FX* versus *FX*‐*R*
Biological							
Hb (g/dl)	13.8 (1.2)	13.8 (1.2)	13.9 (1.2)	0.4	–	–	–
CRP (mg/L)^a^	1.0 [0.5; 2.0]	1.1 [0.5; 2.3]	0.9 [0.5; 2.6]	0.1	–	–	–
eGFR (ml/min/1.73 m²)^a^	94.9 [88.9; 99.1]	92.1 [88.2; 99.1]	93.2 [88.5; 99.2]	0.6			
Sodium (mmol/L)^a^	141.0 [140.0; 142.0]	141.0 [140.0; 142.0]	141.0 [140.0; 141.8]	0.5	–	–	–
Potassium (mmol/L)^a^, ^b^	4.1 [4.0; 4.3]	4.1 [4.0; 4.3]	4.0 [3.9; 4.2]	**0.02**	1.0	1.0	0.3
Uric acid (mg/dl)^a^	5.1 [4.1; 5.9]	1.9 [1.2; 2.2]	0.2 [0.2; 0.3]	**<0.0001**	**<0.0001**	**<0.0001**	**<0.0001**
LDH (U/L)^a^	160.0 [137.3; 172.0]	158.5 [139.8; 168.5]	159.0 [139.3; 170.8]	0.8	–	–	–
ACE (U/L)	34.5 (16.8)	34.2 (15.6)	34.4 (16.2)	0.9	–	–	–
Aldosterone (ng/L)^a^	55.5 [41.1; 75.9]	50.8 [39.9; 68.8]	48.3 [35.3; 64.5]	0.1	–	–	–
PRA (mUI/L)^a^	9.2 [3.9; 13.7]	8.5 [4.4; 17.2]	7.3 [3.8; 13.0]	0.3	–	–	–
Aldosterone/PRA ratio (ng/mUI)^a^	7.9 [4.4; 14.7]	6.4 [3.7; 11.0]	6.8 [4.2; 13.0]	0.8	–	–	–
Angiotensin II (pg/ml)^a^	3.3 [2.1; 5.5]	3.9 [2.1; 5.3]	3.4 [2.0; 6.0]	0.6	–	–	–
Urine protein/creatinine ratio (g/g)^a^	0.06 [0.05; 0.1]	0.06 [0.04; 0.1]	0.06 [0.04; 0.1]	0.1			
Urine sodium/creatinine ratio (mmol/g)^a^	120.2 [69.9; 142.1]	97.1 [68.2; 155.4]	97.6 [57.2; 121.8]	0.2			
Urine uric acid/creatinine ratio (mmol/g)^a^	38.3 [27.2; 50.9]	10.1 [7.1; 11.2]	2.9 [1.9; 5.6]	**<0.0001**	**<0.0001**	**<0.0001**	**<0.0001**
Hemodynamic parameters							
Systolic BP (mmHg)	131 (16)	129 (17)	130 (14)	0.1	–	–	–
Diastolic BP (mmHg)	81 (10)	79 (10)	81 (9)	**0.008**	**0.05**	1.0	**0.03**
PP (mmHg)	51 (10)	50 (10)	49 (8)	0.5	–	–	–
MAP (mmHg)	98 (11)	96 (13)	97 (10)	**0.02**	**0.04**	1.0	0.2
HR (/min)	57 (7)	58 (7)	58 (7)	0.3	–	–	–
AIx (%)	29.6 (7.8)	27.5 (7.8)	28.1 (7.8)	**0.008**	**0.009**	0.1	1.0
AIx75 (%)	20.5 (7.2)	19.1 (7.2)	19.3 (7.2)	**0.03**	0.07	0.1	1.0
cr‐PWV (m/s)	7.0 (0.6)	6.8 (0.6)	6.8 (0.6)	0.2	–	–	–
cf‐PWV (m/s)	7.2 (1.2)	7.2 (1.8)	7.4 (1.8)	0.3	–	–	–

Significant *p* value are in bold.

Abbreviations: P, indicates placebo; FX, febuxostat; FX‐R, febuxostat and rasburicase; Hb, hemoglobin; CRP, C‐reactive protein; eGFR, estimated glomerular filtration rate; LDH, lactate dehydrogenase; ACE, angiotensin‐converting enzyme; PRA, plasma renin activity; BP, blood pressure; PP, pulse pressure; MAP, mean arterial pressure; HR, heart rate; AIx and AIx75, augmentation index and AIx corrected for heart rate; cr‐ and cf‐PWV, carotid‐radial and carotid‐femoral pulse wave velocity.

^a^ not normally distributed.

^b^ *n* =35.

### 3.3 Endothelial function

**FIGURE 2:**
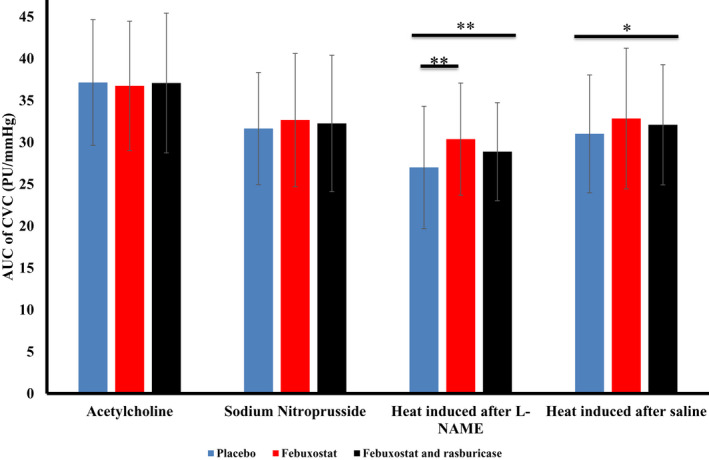
Endothelium function assessment. Figure 2 represents the area under the curve (AUC) of cutaneous vascular conductance (CVC = cutaneous flow in perfusion units/mean arterial pressure in mmHg) according to the stimuli (acetylcholine and sodium nitroprusside iontophoresis or heat after L‐N‐arginine‐methyl‐ester (L‐NAME) and saline iontophoresis) for the three treatments (placebo, febuxostat, and febuxostat and rasburicase). No differences were found under acetylcholine and sodium nitroprusside. Heat‐induced vasodilatation differed according to the treatment received. The difference states between febuxostat and placebo treatments after L‐NAME iontophoresis. * indicates a *p*‐*value* under 0.05 and **, a *p*‐*value* under 0.01

The AUC of CVC induced by ACh‐ and SNP‐iontophoresis were 37.1 (7.5), 36.8 (7.7), 37.1 (8.3); and 31.6 (6.7), 32.7 (8.0), 32.2 (8.1) PU/mmHg for placebo, febuxostat, and febuxostat–rasburicase treatments (ANOVA *p* = 0.9 and 0.5, respectively). After the pre‐treatment with L‐NAME iontophoresis, the AUC of late‐phase CVC induced by heat was 27.0 (7.3), 30.4 (6.7), and 28.9 (5.9) PU/mmHg for placebo, febuxostat, and febuxostat–rasburicase treatments (ANOVA *p* = 0.003; placebo vs. febuxostat *p* = 0.007). Saline iontophoresis resulted in a similar change in the AUC of late‐phase CVC induced by heat: 31.0 (7.0), 32.8 (8.4), and 32.1 (7.2) PU/mmHg for placebo, febuxostat, and febuxostat–rasburicase treatments (ANOVA *p* = 0.04; placebo vs. febuxostat *p* = 0.054) (Figure 2). Carryover effects were not found.

### 3.4 Adverse effects

Hot spells were reported in 2, 10, and 8 participants treated by placebo, febuxostat, and febuxostat–rasburicase, respectively (ANOVA *p* = 0.04; placebo vs. febuxostat *p* = 0.03). Approximately 50% of the participants reported at least one side effect. The most prevalent side effects were headache, abdominal pain, and hot spells. No statistical difference was found between treatments for headache and abdominal pain.

### 3.5 Hemodynamic parameters

Diastolic BP decreased with febuxostat compared to placebo and febuxostat–rasburicase (ANOVA *p* = 0.008, placebo vs. febuxostat *p* = 0.05, and febuxostat vs. febuxostat–rasburicase *p* = 0.03). In addition, MAP declined with febuxostat (ANOVA *p* = 0.02, placebo vs. febuxostat *p* = 0.04). Furthermore, there was a decrease in AIx with febuxostat compared to placebo (ANOVA *p* = 0.008, placebo vs. febuxostat *p* = 0.009). An identical trend was observed for AIx75; however, this was not significant upon multiple comparison (ANOVA *p* = 0.03). There was no significant difference in HR, cr‐, and cf‐PWV between treatments (Table 2). There were no carryover effects.

### 3.6 Markers of oxidative stress and endothelial function

Allantoin decreased with febuxostat and increased with febuxostat–rasburicase (Friedman and multiple comparisons *p* < 0.0001). FRAP decreased and SOD activity increased with febuxostat and febuxostat–rasburicase (ANOVA and multiple comparisons *p* < 0.0001; and Friedmann and placebo vs. febuxostat and placebo vs. febuxostat–rasburicase *p* < 0.0001, respectively). The MPO concentration did not differ between treatments in contrast to MPO activity, which was profoundly reduced with febuxostat–rasburicase (Friedman, placebo vs. febuxostat–rasburicase and febuxostat vs. febuxostat–rasburicase *p* < 0.0001). Homocitrulline/lysine and chlorotyrosine/tyrosine ratios and MDA concentration did not differ between treatments. No significant differences were found for the endothelial markers sICAM‐1, sP‐selectin, and nitrites. Additionally, there were no carryover effects. UA levels positively correlated with MPO activity and FRAP (Spearman's coefficient 0.6 and 0.95, both *p* < 0.0001, respectively) and negatively correlated with allantoin and SOD activity (Spearman's coefficient −0.5 and −0.4, both *p* < 0.0001, respectively) (Table 3). IL‐6 and IL‐8 levels were under the detection range (data not shown).

**TABLE 3:** Markers of oxidative stress and endothelial function

Measures	Placebo (*n* = 36)	Febuxostat (*n* = 36)	Febuxostat and Rasburicase (*n* = 36)	*p*			
				*ANOVA*	*P* versus *FX*	*P* versus *FX*‐*R*	*FX* versus *FX*‐*R*
Oxidative stress							
Allantoin (µmol/L)^a^	0.6 [0.5; 0.8]	0.5 [0.4; 0.6]	20.3 [13.1; 27.8]	**<0.0001**	**<0.0001**	**<0.0001**	**<0.0001**
Chloro‐Tyrosine/Tyrosine ratio (x 10^−5^)^a^	6.2 [5.3; 6.9]	6.5 [5.5; 7.0]	6.7 [5.3; 7.4]	0.7	–	–	–
Homocitrulline/Lysine ratio (x 10^−5^)^a^	112.1 [98.0; 128.3]	114.5 [100.1; 134.4]	117.8 [95.6; 135.3]	0.6	–	–	–
MPO activity (mU/ml)	1.0 [0.8; 1.3]	0.9 [0.6; 1.2]	0.2 [0.1; 0.5]	**<0.0001**	0.8	**<0.0001**	**<0.0001**
MPO (ng/ml)^a^, ^b^	122.0 [86.7; 177.5]	144.8 [74.6; 175.4]	144.9 [89.1; 205.1]	0.6	–	–	–
MDA (nmol/mL)^a^	1.4 [1.2; 2.1]	1.3 [1.2; 2.4]	1.3 [1.2; 1.9]	0.1	–	–	–
FRAP (µmol/L)	1335.6 (264.0)	836.5 (162.6)	594.1 (81.0)	**<0.0001**	**<0.0001**	**<0.0001**	**<0.0001**
SOD activity (mU/ml)^a^, ^b^	0.6 [0.5; 0.6]	0.7 [0.6; 0.8]	0.7 [0.6; 0.7]	**<0.0001**	**<0.0001**	**<0.0001**	0.4
Endothelial function							
Nitrites (µmol/L)^a^	4.8 [4.4; 5.6]	4.9 [4.4; 5.4]	4.8 [4.5; 5.7]	0.2	–	–	–
sICAM−1 (ng/ml)^a^	198.1 [175.2; 225.6]	198.1 [170.0; 233.5]	196.4 [177.3; 222.8]	0.8	–	–	–
sP‐selectin (ng/ml)^a^	25.2 [18.2; 32.1]	24.2 [18.3; 29.6]	27.0 [18.7; 31.8]	0.4	–	–	–

Significant *p* value are in bold.

Abbreviations: P, indicates placebo; FRAP, Ferric reducing antioxidant power; FX, febuxostat; FX‐R, febuxostat and rasburicase; MDA, malondialdehyde; MPO, myeloperoxidase; SOD, superoxide dismutase; sICAM‐1, soluble InterCellular Adhesion Molecule 1.

^a^ not normally distributed.

^b^ n = 35.

### 3.7 Subgroups analyses

Detailed analysis for subgroups can be found in Supplementary Materials.

## 4 DISCUSSION

The main new findings of this study are first, that the combination of febuxostat and rasburicase is more effective than febuxostat alone to drastically and rapidly reduce the UA concentration regardless of gender, age, BP, or UA concentration at baseline. Second, the microvascular response to heat improves when the concentration of UA is moderately reduced. Third, diastolic BP, MAP, and aortic stiffness decrease in association with moderately reduced UA levels. Finally, an acute and extreme decrease in UA leads to a decreased plasma antioxidant capacity as well as reduced MPO activity. The present study complete our first work (De Becker, Coremans, et al., 2019).

### 4.1 Effects on endothelial function

As in our previous study (De Becker, Coremans, et al., 2019), an acute reduction in UA levels did not impact hyperemia induced by ACh or SNP iontophoresis. In addition, the concentrations of nitrites, sICAM‐1, and sP‐selectin did not change between treatments. The acute effect of urate‐lowering therapies on endothelial function has not been studied before our works. Long‐term allopurinol improved endothelial function assessed by flow‐mediated dilation (Alem, 2018; Kanbay et al., 2014). Previous humans trials with febuxostat showed either a reduction in ADMA (an endothelial dysfunction marker) in hemodialyzed patients treated for 2 months or no effects on coronary endothelial function assessed by magnetic resonance imaging in patients with coronary artery disease after 6 weeks (Hays et al., 2018). The positive effect of allopurinol on endothelial function is potentially unrelated to XO inhibition and ROS reduction due to its inherent antioxidant properties (Minor et al., 1993). Conversely, febuxostat is a more powerful and complete ROS production blocker as it inhibits both isoforms of xanthine oxidoreductase (Cicalese et al., 2019). Long‐term effects of febuxostat alone and in combination with rasburicase on endothelial function should be investigated.

Responses to heat were improved by moderate hypouricemia after L‐NAME and saline iontophoresis with a more pronounced effect when NOS was blocked. This suggests that NO does not play a role in the improvement of the response to heat when UA is reduced and that variations in UA levels influence other mechanisms involved in endothelial response to heat. Taken together with data from ACh and SNP iontophoresis, it is thus very likely that the NO pathway is not disturbed by hypouricemia *per se*. Heat‐induced hyperemia involved several complex mechanisms such as NO, neurotransmitters, and endothelium‐derived hyperpolarizing factors (EDHF) and probably other actors. Among them, EDHF do not play a major role in microvascular function in healthy circumstances (Félétou & Vanhoutte, 2006) but becomes an important rescue system in case of reduced NO bioavailability (Félétou, 2011). In our study, we observed that an acute and moderate hypouricemia restored slightly thermal vasodilatation especially in the presence of NOS inhibition. We interpret this as a favorable effect, although more experience is needed to confirm our hypothesis especially since the combination of febuxostat and rasburicase mitigated this modest improvement.

### 4.2 Effects on blood pressure and arterial stiffness

The main effect of acute UA reduction on BP was observed with febuxostat, with a 2 mmHg reduction in diastolic BP compared to placebo and febuxostat–rasburicase. Identical results were observed for MAP, AIx, and AIx75, which decreased with febuxostat. There were no differences in PWV according to treatments. The discrepancies between measures of arterial stiffness could be explained by the fact that AIx depends on the propagation rate of pressure wave in the arterial tree but also on the reflected wave by the peripheral arterial system. Despite an excellent correlation between AIx and PWV, AIx is potentially more influenced by blood pressure (Yasmin, 1999). These aspects could explain the absence of change in PWV in our study. We did not observe a significant difference in RAS activity in our entire study population; however, normotensive participants had a decrease in aldosterone levels in conjunction with UA reduction. Despite the lack of significance, a lower RAS activity remains a plausible explanation for the decrease in BP with UA level reduction. Females presented higher AIx than males. This finding has been previously reported; however, it has not yet been completely explained (Mitchell et al., 2004).

The effect of hypouricemia on hemodynamic parameters only occurs in participants without hypertension. The vascular system does not respond to UA variation when hypertension is settled. The presence of antihypertensive medications blocking the RAS in these patients may have played a role in this observation. Among the trials studying the effect of urate‐lowering therapies on BP, positive results were observed in hypertensive children, though rarely in adults (Gois and Erdm, 2017). Therefore, the vascular system also appears to be less responsive to UA variation with age. Previous studies on adults are rare and demonstrated the controversial effect of febuxostat on BP, arterial stiffness, and RAS (Gunawardhana et al., 2017; Lytvyn et al., 2017; Ohta et al., 2017; Tani et al., 2015; Tausche et al., 2014; Sezai et al., 2015). The effects of recombinant uricase have rarely been studied. Acute administration of uricase did not affect BP or AIx in type 1 diabetes patients (Waring et al., 2007); however, recurrent infusion of uricase (fortnightly) reduced BP in patients with gout (Johnson et al., 2019).

### 4.3 Effects on biological data, oxidative stress, and inflammation

Allantoin is a product of the reaction of UA and other major oxidant products or enzymes such as MPO (Meotti et al., 2011). We observed a reduction in allantoin with febuxostat, which is likely related to a reduction of substrate (UA) than oxidative stress. Rasburicase, which transforms UA into allantoin, primarily increases allantoin concentration. The acute effects of UA reduction on oxidative products are not significant but warrant interest; for example, MDA levels decreased with febuxostat and with febuxostat–rasburicase. Febuxostat reduced the plasma antioxidant capacity by approximately 37%, whereas the addition of rasburicase reduced the FRAP by approximately 55%. The decrease of FRAP is directly linked to the loss of UA in plasma. SOD activity was also impacted by the treatments and increased with febuxostat and with febuxostat–rasburicase. The increase of SOD activity is the result of ROS reduction through XO inhibition by febuxostat. The addition of rasburicase does not improve or reverse this effect. Hyperuricemia is associated with high levels of MDA and low levels of SOD in asymptomatic patients of approximately 55 years of age (Zhou et al., 2018). We confirmed this association by the blockade of XO. A reduction in MDA and an increase in SOD were previously described in hemodialyzed patients after 2 months of febuxostat (Alshahawey et al., 2017). Allopurinol is known to reduce MDA levels (Higgins et al., 2011). The MPO concentration and activity were differently affected by febuxostat, and febuxostat and rasburicase. Interestingly, the MPO concentration did not differ whereas MPO activity was profoundly reduced by febuxostat–rasburicase. The severe hypouricemia in plasma is related to a reduced intracellular UA concentration and, therefore, a lower oxidant pressure inside cells. As a result, even in the event of an unaltered release of MPO (identical concentration), MPO activity is reduced. Our study confirms that XO blockade and UA reduction rapidly affect the oxidative balance, even following a few hours, with a variation of the plasma antioxidant capacity. The effects on antioxidant actors appear before the effects on oxidants or oxidative products. Again, the variation in the UA concentration faced an opposite effect depending on the location. A severe reduction in UA concentration is associated with a decreased plasma antioxidant capacity (potentially harmful), as well as reduced oxidant pressure inside cells with diminished MPO activity (potentially beneficial).

Our work presents several limitations. We only study the acute effect and our measures were taken shortly after intervention. Added measures after the intervention could have afforded more precision to this work. Although the number of subjects in the present is not considerable, this is mitigated by a strict crossover design and sufficient power according to the sample size calculation. Furthermore, our findings are restricted to adults with a median age of 58 years, of which a number were hypertensives. L‐NAME is a non‐selective inhibitor of all three NO synthase isoforms and thus, not specific for endothelial NO synthase. L‐NAME iontophoresis may offer less stronger inhibition of NO than with transdermal microdialysis which is invasive and not assessed in our study. The limited contribution of NO synthase in the thermal vasodilatation we observed is most likely because middle‐aged adults and elderly are less responsive to heat‐induced hyperemia and less L‐NAME respondent because of naturally reduced NO bioavailability, also reported upon microdialysis (Bruning et al., 2012). Finally, hypertensive patients were studied under their standard medication, because treatment withdrawal could potentially affect the investigated parameters, as well as for evident ethical reasons.

Acute reduction in UA levels has limited beneficial and harmful consequences to the cardiovascular system. In contrast to severe hypouricemia, acute moderate UA reduction in middle‐aged adults encompasses slight improved endothelial function, modest BP, and arterial stiffness reduction. While XO inhibition increases antioxidant defense by SOD, plasma antioxidant capacity is strongly reduced impairing the oxidative balance. Concurrently, severe hypouricemia is also accompanied by a decrease in MPO activity.

## CONFLICT OF INTEREST

DE BECKER Benjamin; HUPKENS Emeline; DEWACHTER Laurence; COREMANS Catherine; DELPORTE Cédric; VAN ANTWERPEN Pierre; FRANCK Thierry; ZOUAOUI BOUDJELTIA and CULLUS Pierre declare they have no conflict of interest. VAN DE BORNE Philippe's employer received honoraria for lecturing/advisory boards from Amgen, Bayer, Boehringer‐Ingelheim, Daïchi‐Sankyo, Idorsia, Menarini, Novo Nordisk, Sanofi.

## AUTHORS CONTRIBUTIONS

B.D.B and Ph.v.d.B designed the study. B.D.B recruited, collected, and analyzed the data. B.D.B, C.C, E.H performed the biological measures of oxidative stress under the supervision of L.D, T.F, C.D, K.Z, and P.v.A. Statistical analysis was discussed with P.C. B.D.B and Ph.v.d.B wrote the article and all authors approved the final version.

## Supporting information



Supplementary Material

## References

[phy215018-bib-0001] Adamopoulos, D., Argacha, J.‐F., Gujic, M., Preumont, N., Degaute, J.‐P., & van de Borne, P. (2009). Acute effects of nicotine on arterial stiffness and wave reflection in healthy young non‐smokers. Clinical and Experimental Pharmacology and Physiology, 36(8), 784–789. 10.1111/j.1440-1681.2009.05141.x 19207722

[phy215018-bib-0002] Agarwal, V., Hans, N., & Messerli, F. H. (2013). Effect of allopurinol on blood pressure: a systematic review and meta‐analysis. The Journal of Clinical Hypertension, 15(6), 435–442. 10.1111/j.1751-7176.2012.00701.x 23730993 PMC8033809

[phy215018-bib-0003] Alem, M. (2018). Allopurinol and endothelial function: A systematic review with meta‐analysis of randomized controlled trials. Cardiovascular Therapeutics, 36(4):e12432. 29673103 10.1111/1755-5922.12432 PMC6175067

[phy215018-bib-0004] Alshahawey, M., Mahmoud Shahin, S., Wahid Elsaid, T., & Ali, S. N. (2017). Effect of Febuxostat on the endothelial dysfunction in hemodialysis patients: A randomized, placebo‐controlled, double‐blinded study. American Journal of Nephrology, 45, 452–459. 28463849 10.1159/000471893

[phy215018-bib-0005] Borghi, C., Tykarski, A., Widecka, K., Filipiak, K. J., Domienik‐Karłowicz, J., Kostka‐Jeziorny, K., Varga, A., Jaguszewski, M., Narkiewicz, K., & Mancia, G. (2018). Expert consensus for the diagnosis and treatment of patient with hyperuricemia and high cardiovascular risk. Cardiology Journal, 25(5), 545–564. 10.5603/CJ.2018.0116 30394510

[phy215018-bib-0006] Bruning, R. S., Santhanam, L., Stanhewicz, A. E., Smith, C. J., Berkowitz, D. E., Kenney, W. L., & Holowatz, L. A. (2012). Endothelial nitric oxide synthase mediates cutaneous vasodilation during local heating and is attenuated in middle‐aged human skin. Journal of Applied Physiology, 112, 2019–2026. 22500004 10.1152/japplphysiol.01354.2011 PMC3378394

[phy215018-bib-0007] Cicalese, S., Scalia, R., & Eguchi, S. (2019). Xanthine oxidase inhibition as a potential treatment for aortic stiffness in hypertension. American Journal of Hypertension, 32(3), 234–236. 30561498 10.1093/ajh/hpy197 PMC6371954

[phy215018-bib-0008] Cicero, A. F. G., Pirro, M., Watts, G. F., & Mikhailidis, D. P. (2017). Effects of allopurinol on endothelial function: A systematic review and meta‐analysis of randomized placebo‐controlled trials. Drugs, 78(1), 99–109. 10.1007/s40265-017-0839-5 29139092

[phy215018-bib-0009] De Becker, B., Borghi, C., Burnier, M., & van de Borne, P. (2019). Uric acid and hypertension: a focused review and practical recommendations. Journal of Hypertension, 37(5), 878–883. 10.1097/HJH.0000000000001980. 30620339

[phy215018-bib-0010] De Becker, B. , Coremans, C., Chaumont, M., Delporte, C., Van Antwerpen, P. , Franck, T., Rousseau, A., Zouaoui Boudjeltia, K., Cullus, P., & van de Borne, P. (2019). Severe hypouricemia impairs endothelium‐dependent vasodilatation and reduces blood pressure in healthy young men: A randomized, placebo‐controlled, and crossover study. Journal of the American Heart Association, 8(23), e013130. 31752638 10.1161/JAHA.119.013130 PMC6912967

[phy215018-bib-0011] Delporte, C., Franck, T., Noyon, C. Dufour, D., Rousseau, A., Madhoun, P., Desmet, J‐M., Serteyn, D., Raes, M., Nortier, J., Vanhaeverbeek, M., Moguilevsky, N., Nève, J., Vanhamme, L., Van Antwerpen, P., & Zouaoui Boudjeltia, K. (2012). Simultaneous measurement of protein‐bound 3‐chlorotyrosine and homocitrulline by LC‐MS/MS after hydrolysis assisted by microwave: Application to the study of myeloperoxidase activity during hemodialysis. Talanta, 99, 603–609. 10.1016/j.talanta.2012.06.044. 22967600

[phy215018-bib-0012] Deng, G., Qiu, Z., Li, D., Fang, Y., & Zhang, S. (2016). Effects of allopurinol on arterial stiffness: A Meta‐analysis of randomized controlled trials. Medical Science Monitor, 22, 1389–1397. 27110924 10.12659/MSM.898370 PMC4847559

[phy215018-bib-0013] Dreyfuss, C., Wauters, A., Adamopoulos, D., Pochet, S., Azarkan, M., Berkenboom, G., van de Borne, P., & Argacha, J. F. (2013). L‐NAME iontophoresis: A tool to assess NO‐mediated vasoreactivity during thermal hyperemic vasodilation in humans. Journal of Cardiovascular Pharmacology, 61(5), 361–368. 10.1097/FJC.0b013e3182858f81. 23318989

[phy215018-bib-0014] Félétou, M. (2011). The endothelium part 2: EDHF‐mediated Responses “The Classical Pathway”. Morgan & Claypool Life Sciences Publishers Series INTEGRATED. 21850764

[phy215018-bib-0015] Félétou, M., & Vanhoutte, P. M. (2006). Endothelium‐derived hyperpolarizing factor where are we now? Arteriosclerosis, Thrombosis, and Vascular Biology, 26, 1215–1225. 10.1161/01.ATV.0000217611.81085.c5 16543495

[phy215018-bib-0016] Franck, T., Minguet, G., Delporte, C., Derochette, S., Zouaoui Boudjeltia, K., Van Antwerpen, P., Gach, O., Deby‐Dupont, G., Mouithys‐Mickalad, A., & Serteyn, D. (2015). An immunological method to combine the measurement of active and total myeloperoxidase on the same biological fluid and its application in finding inhibitors which interact directly with the enzyme. Free Radical Research, 49(6), 790–799. 10.3109/10715762.2015.1027197 25968947

[phy215018-bib-0017] Gois, P., & Erdm, S. (2017). Pharmacotherapy for hyperuricemia in hypertensive patients (Review). Cochrane Database Systematic Review, 4(4), CD008652. 10.1002/14651858.CD008652.pub3 PMC6478066 28406263

[phy215018-bib-0018] Grayson, P. C., Young Kim, S., Lavalley, M., & Choi, H. K. (2011). Hyperuricemia and incident hypertension: A systematic review and meta‐analysis. Arthritis Care & Research, 63(1), 102–110. 10.1002/acr.20344. 20824805 PMC3016454

[phy215018-bib-0019] Gunawardhana, L., McLean, L., Punzi, H. A., Hunt, B., Palmer, R. N., Whelton, A., & Feig, D. I. (2017). Effect of Febuxostat on ambulatory blood pressure in subjects with hyperuricemia and hypertension: A phase 2 randomized placebo‐controlled study. Journal of the American Heart Association, 6(11), e006683. 29102979 10.1161/JAHA.117.006683 PMC5721765

[phy215018-bib-0020] Hays, A. G., Iantorno, M., Schär, M., Lai, S., Czarny, M., Breton, E., Palmer, R. N., Whelton, A., Weiss, R. G., & Gerstenblith, G. (2018). The influence of febuxostat on coronary artery endothelial dysfunction in patients with coronary artery disease: A phase. American Heart Journal, 197, 85–93. 29447788 10.1016/j.ahj.2017.11.006

[phy215018-bib-0021] Higgins, P., Dawson, J., Lees, K. R., Mcarthur, K., Quinn, T. J., & Walters, M. R. (2011). Xanthine oxidase inhibition for the treatment of cardiovascular disease: A systematic review and meta‐analysis. Cardiovascular Therapeutics, 30(4), 217–226. 22099531 10.1111/j.1755-5922.2011.00277.x

[phy215018-bib-0022] Johnson, J. M., & Kellogg, D. L. (2010). Local thermal control of the human cutaneous circulation. Journal of Applied Physiology, 109(4), 1229–1238. 10.1152/japplphysiol.00407.2010 20522732 PMC2963328

[phy215018-bib-0023] Johnson, R. J., Choi, H. K., Yeo, A. E., Lipsky, P. E., & Commentary, S. E. (2019). Uric acid pegloticase treatment significantly decreases blood pressure in patients with chronic gout. Hypertension, 74, 95–101. 31079535 10.1161/HYPERTENSIONAHA.119.12727

[phy215018-bib-0024] Johnson, R. J., Feig, D. I., Herrera‐Acosta, J., & Kang, D.‐H. (2005). Resurrection of uric acid as a causal risk factor in essential hypertension. Hypertension, 45(1), 18–20. 10.1161/01.HYP.0000150785.39055.e8 15557387

[phy215018-bib-0025] Johnson, R. J., Sánchez‐Lozada, L. G., Mazzali, M., Feig, D. I., Kanbay, M., & Sautin, Y. Y. (2013). What are the key arguments against uric acid as a true risk factor for hypertension? Hypertension, 61(5), 948–951. 23460277 10.1161/HYPERTENSIONAHA.111.00650

[phy215018-bib-0026] Kamel, B., Graham, G. G., Williams, K. M., Pile, K. D., & Day, R. O. (2016). Clinical pharmacokinetics and pharmacodynamics of febuxostat. Clinical Pharmacokinetics, 56(5), 459–475. 10.1007/s40262-016-0466-4 27753003

[phy215018-bib-0027] Kanbay, M., Segal, M., Afsar, B., Kang, D.‐H., Rodriguez‐Iturbe, B., & Johnson, R. J. (2013). The role of uric acid in the pathogenesis of human cardiovascular disease. Heart, 99(11), 759–766. 10.1136/heartjnl-2012-302535. 23343689

[phy215018-bib-0028] Kanbay, M., Siriopol, D., Nistor, I. et al (2014). Effects of allopurinol on endothelial dysfunction: A meta‐analysis. American Journal of Nephrology, 39(4), 348–356. 10.1159/000360609 24751886

[phy215018-bib-0029] Khosravan, R., Grabowski, B. A., Wu, J., Joseph‐ridge, N., & Vernillet, L. (2006). Pharmacokinetics, pharmacodynamics and safety of febuxostat, a non‐purine selective inhibitor of xanthine oxidase, in a dose escalation study in healthy subjects. Clinical Pharmacokinetics, 45(8), 821–841. 10.2165/00003088-200645080-00005 16884320

[phy215018-bib-0030] Kubli, S., Waeber, B., Dalle‐Ave, A., & Feihl, F. (2000). Reproducibility of laser doppler imaging of skin blood flow as a tool to assess endothelial function. Journal of Cardiovascular Pharmacology, 36(5), 640–648. 10.1097/00005344-200011000-00014 11065225

[phy215018-bib-0031] Levey, A. S., Stevens, L. A., Schmid, C. H., Zhang, Y., Castro, A. F., Feldman, H. I., Kusek, J. W., Eggers, P., Van Lente, F., Greene, T., & Coresh, J. (2009). A new equation to estimate glomerular filtration rate. Annals of Internal Medicine, 150(9), 604–612. 10.7326/0003-4819-150-9-200905050-00006 19414839 PMC2763564

[phy215018-bib-0032] Lytvyn, Y., Har, R., Locke, A., Lai, V., Fong, D., Advani, A., Perkins, B. A., & Cherney, D. Z. I. (2017). Renal and vascular effects of uric acid lowering in normouricemic patients with uncomplicated type 1 diabetes. Diabetes, 66(7), 1939–1949. 28408434 10.2337/db17-0168

[phy215018-bib-0033] Mazzali, M., Kanbay, M., Segal, M. S., Shafiu, M., Jalal, D., Feig, D. I., & Johnson, R. J. (2010). Uric acid and hypertension: Cause or effect? Current Rheumatology Reports, 12(2), 108–117. 20425019 10.1007/s11926-010-0094-1

[phy215018-bib-0034] Meotti, F. C., Jameson, G. N. L., Turner, R. Harwood, D. T., Stockwell, S., Rees, M. D., Thomas, S. R., & Kettle, A. J. (2011). Urate as a physiological substrate for myeloperoxidase. Journal of Biological Chemistry, 286(15), 12901–12911. 10.1074/jbc.M110.172460 PMC3075637 21266577

[phy215018-bib-0035] Minor, T., Isselhard, W., Yamamoto, Y., Obara, M., & Saad, S. (1993). The effects of allopurinol and SOD on lipid peroxidation and energy metabolism in the liver after ischemia in an aerobic/anaerobic persufflation. Surgery Today, 23, 728–732. 10.1007/BF00311713 8400677

[phy215018-bib-0036] Mitchell, G. F., Hwang, S., Vasan, R. S. Larson, M. G., Pencina, M. J., Hamburg, N. M., Vita, J. A., Levy, D., & Benjamin, E. J. (2010). Arterial stiffness and cardiovascular events the framingham heart study gary. Circulation, 121, 505–511. 10.1161/CIRCULATIONAHA.109.886655 20083680 PMC2836717

[phy215018-bib-0037] Mitchell, G. F., Parise, H., Benjamin, E. J., Larson, M. G., Keyes, M. J., Vita, J. A., Vasan, R. S., & Levy, D. (2004). Changes in arterial stiffness and wave reflection with advancing age in healthy men and women the framingham heart study. Hypertension, 43, 1239–1245. 10.1161/01.HYP.0000128420.01881.aa 15123572

[phy215018-bib-0038] Ohta, Y., Ishizuka, A., Arima, H. Hayashi, S., Iwashima, Y., Kishida, M., Yoshihara, F., Nakamura, S., & Kawano, Y. (2017). Effective uric acid‐lowering treatment for hypertensive patients with hyperuricemia. Hypertension Research, 40(3), 259–263. 10.1038/hr.2016.139 27760998

[phy215018-bib-0039] Perez‐Gomez, M. V., Bartsch, L., Castillo‐Rodriguez, E., Fernandez‐Prado, R., Kanbay, M., & Ortiz, A. (2019). Potential dangers of serum urate‐lowering therapy. American Journal of Medicine, 132(4), 457–467. 10.1016/j.amjmed.2018.12.010 30611833

[phy215018-bib-0040] Qu, L., Jiang, H., & Chen, J. (2016). Effect of uric acid‐lowering therapy on blood pressure: systematic review and meta‐analysis Li‐hui. Annals of Medicine, 49(2), 142–156. 27689859 10.1080/07853890.2016.1243803

[phy215018-bib-0041] Sezai, A., Soma, M., Nakata, K., Nakata, K‐i., Osaka, S., Ishii, Y., Yaoita, H., Hata, H., & Shiono, M. (2015). Comparison of febuxostat and allopurinol for hyperuricemia in cardiac surgery patients with chronic kidney disease (NU‐FLASH trial for CKD). Journal of Cardiology, 66(4), 298–303. 10.1016/j.jjcc.2014.12.017 25649025

[phy215018-bib-0042] Tani, S., Nagao, K., & Hirayama, A. (2015). Effect of febuxostat, a xanthine oxidase inhibitor, on cardiovascular risk in hyperuricemic patients with hypertension: A prospective, open‐label, pilot study. Clinical Drug Investigation, 35(12), 823–831. 10.1007/s40261-015-0349-8 26482071

[phy215018-bib-0043] Tausche, A. K., Christoph, M., Forkmann, M., Richter, U., Kopprasch, S., Bielitz, C., Aringer, M., & Wunderlich, C. (2014). As compared to allopurinol, urate‐lowering therapy with febuxostat has superior effects on oxidative stress and pulse wave velocity in patients with severe chronic tophaceous gout. Rheumatology International, 34(1), 101–109. 10.1007/s00296-013-2857-2 24026528

[phy215018-bib-0044] Ueng, S. (2005). Rasburicase (Elitek): a novel agent for tumor lysis syndrome. Baylor University Medical Center Proceedings, 18, 275–279. 10.1080/08998280.2005.11928082 16200184 PMC1200736

[phy215018-bib-0045] Verdecchia, P., Schillaci, G., Reboldi, G., Santeusanio, F., Porcellati, C., & Brunetti, P. (2000). Relation between serum uric acid and risk of cardiovascular disease in essential hypertension: The PIUMA study. Hypertension, 36(6), 1072–1078. 10.1161/01.HYP.36.6.1072 11116127

[phy215018-bib-0046] Waring, W. S., McKnight, J. A., Webb, D. J., & Maxwell, S. R. J. (2007). Lowering serum urate does not improve endothelial function in patients with type 2 diabetes. Diabetologia, 50(12), 2572–2579. 10.1007/s00125-007-0817-7 17928991

[phy215018-bib-0047] Wauters, A., Dreyfuss, C., Pochet, S. Hendrick, P., Berkenboom, G., van de Borne, P., & Argacha, J‐F. (2013). Acute exposure to diesel exhaust impairs nitric oxide‐mediated endothelial vasomotor function by increasing endothelial oxidative stress. Hypertension, 62(2), 352–358. 10.1161/HYPERTENSIONAHA.111.00991 23798345

[phy215018-bib-0048] Yasmin, B. M. J. (1999). Similarities and differences between augmentation index and pulse wave velocity in the assessment of arterial stiffness. QJM, 92(10), 595–600. 10.1093/qjmed/92.10.595 10627881

[phy215018-bib-0049] Yiginer, O., Ozcelik, F., Inanc, T., Aparci, M., Ozmen, N., Cingozbay, B. Y., Kardesoglu, E., Suleymanoglu, S., Sener, G., & Cebeci, B. S. (2008). Allopurinol improves endothelial function and reduces oxidant‐inflammatory enzyme of myeloperoxidase in metabolic syndrome. Clinical Research in Cardiology, 97(5), 334–340. 10.1007/s00392-007-0636-3 18330493

[phy215018-bib-0050] Zhou, Y., Zhao, M., Pu, Z., Xu, G., & Li, X. (2018). Relationship between oxidative stress and inflammation in hyperuricemia. Medicine, 97(49), e13108. 30544373 10.1097/MD.0000000000013108 PMC6310523

