# Supplementary material for: Acute effects of hypouricemia on endothelium, oxidative stress, and arterial stiffness: A randomized, double‐blind, crossover study
Source: Physiol Rep. 2021 Aug 26;9(17):e15018. doi: 10.14814/phy2.15018 (PMC8387791; doi:10.14814/phy2.15018)
Supplement: Supplementary file 1 — Supplementary Material [file PHY2-9-e15018-s001.docx]

**DATA SUPPLEMENT**

1. **Detailed methods**

**Plan of a session**

Each participant had three sessions, each session lasted four days. On day 1 and 2, subjects were at home. On day 3 and 4 the volunteers came in the clinical research centre. The participants stayed 45 min on day 3 and 2h30 on day 4 in the research facility.

| **Day 1** | **Day 2** | **Day 3** | **Day 4** |
| --- | --- | --- | --- |
| 3 pills orally : placebo or febuxostat 240 mg | 3 pills orally : placebo or febuxostat 240 mg | 3 pills orally : placebo or febuxostat 240 mg +  Intravenous infusion of 0.9% saline or 3 mg of rasburicase for 30 minutes | Measurements |

**Homocitrulline and 3-chlorotyrosine measures** [1]

Acid hydrolysis of proteins

We defrosted plasmas at room temperature.

In a 3 mL hydrolysis quartz vial, we added 20 µL of vortexed plasma, 10 µL of internal standard (^13^C_2_ Lysine [3.36 µM] and ^13^C_9_ Tyrosine [33.6 µM]) and 200 µL of solvent (HCl 6N supplemented with phenol 0.05% [to prevent halogenations of Tyrosine]). Acid hydrolyses using a StartS microwave oven and a protein hydrolysis reactor (Milestone, Italy), was carried out by heating to 110°C for 5 min and maintaining the temperature at 110°C for 30 min and ventilate for 25 min. A temperature sensor was connected to the microwave software in order to set the microwave power. After cooling (30 min), sample solutions were transferred from vials to 2 mL Eppendorf flasks. Vials were washed out with 300 µL of methanol and this latter were added to the Eppendorf flasks. Sample solutions were dried under nitrogen, then centrifugated to dry with liquid nitrogen for 30 minutes.

Amino acids derivatization

Dried samples were derivatized by butanolic-HCl (ultrasonic cleaning for 20 minutes) in a water bath at 65°C for 15 minutes and vortexed after 3 minutes. Excess of butanolic-HCl was evaporated under nitrogen. The samples were diluted in 1 mL formic acid 0.1% and 10 µL were injected to LC-MS/MS.

**Allantoin measure** [2]

Calibration curve

A predefined volume of allantoin solution (concentration from 0.4 to 400 µM) with 10 µL of internal standard (^13^C_2_C_2_H_6_^15^N_4_O_3_ 5 µM in acetonitrile) was added to a predefined volume of water MilliQ and 440 µL of acetonitrile. The obtained concentrations of allantoin were 0.05, 0.1, 0.5, 1, 5, 10, 20, 40, 100, 200 and 400 µM for 40 µL of solution.

Purification of plasma

Plasma samples were defrosted at room temperature, then vortexed and centrifugated at 2500 x g for 3 minutes at room temperature. 40 µL of supernatant was collected and put in 1.5 mL Eppendorf flasks. In these Eppendorf flasks, we added 10 µL of internal standard, 10 µL of water MilliQ and 440 µL of acetonitrile. Samples were mixed and centrifugated at 15000 x g for 15 min at room temperature. We collected 400 µL of supernatant and 20 µL were injected to LC-MS/MS analysis (positive ESI).

**MPO activity :** SIEFED method [3]

Samples preparation

Plasma samples were defrosted at room temperature, then vortexed. 50 µL of plasma was collected and put in a 5 mL vial. 450 µL of buffer was added to get a 1/10 dilution.

Standards: MPO standard solutions were prepared from purified human active MPO as follow blank (0 mU/mL), 0,78 mU/mL, 1,56 mU/mL, 3,13 mU/mL, 6,25 mU/mL, 12,5 mU/mL, 25 and 50 mU/mL.

Procedure

100 µL of each diluted sample and standards were added to the pre-coated plaque and incubated at 37°C for 2hours. The analysis was performed in duplicate. The plaques were washed 4 times with PBS/Tween buffer. Then revelation was performed with the addition of 10 µL of Sodium Nitrite and 100 µL of Amplex Red solution (with phosphate buffer and H_2_O_2_ mM). The plaques were read in the Fluoroskan Ascent FL (Labsystems®) with a kinetic of 30 minutes (Ascent Software 2.6, Thermo Labsystems®).

**List of the used assays**

- MPO : ELISA kit (R&D systems, Minneapolis, MN, USA)

- IL-6 and IL-8 : ELISA kit (BD Biosciences, Franklin Lakes, NJ and ThermoFisher Scientific, Waltham, MA, USA)

- MDA : ELISA kit (MyBiosource, San Diego, CA, USA)

- SOD and Ferric Antioxidant Status Detection Kit (Cu/Zn, Mn, and Fe-SOD, ThermoFisher Scientific, Waltham, MA, USA and FRAP, ThermoFisher Scientific, Waltham, MA, USA).

- sICAM-1 and sP-selectin : ELISA kit (R&D systems, Minneapolis, MN, USA)

- Nitrites measurement (ThermoFisher Scientific, Waltham, MA, USA)

- Angiotensin II : ELISA kit (Sigma-Aldrich, St. Louis, MO, USA).

1. **Supplemental data**

*Subgroups analyses results (see* ***Tables S1.1 to S2.2****)*

Sex

UA and aldosterone levels were higher in males than in females with all treatments. UA concentrations decreased for males and females with febuxostat and with febuxostat and rasburicase (all *p* < 0.0001). BP was mainly higher in males than in females (*p* < 0.05 for diastolic BP).

Higher AIx and AIx75 values were observed in females. There was no difference observed between both sexes for PWV. Diastolic BP decreased in males with febuxostat compared to placebo (*p* = 0.03). A reduction in AIx and AIx75 with febuxostat and with febuxostat and rasburicase was observed in females (ANOVA *p* = 0.001 and 0.02; placebo *vs*. febuxostat *p* = 0.004 and 0.05; placebo *vs*. febuxostat and rasburicase *p* = 0.05, respectively).

Endothelial response to heat was improved by febuxostat after L-NAME iontophoresis in males and after saline iontophoresis in females (*p* < 0.05).

No new changes were found for oxidative stress markers. Overall, males had higher concentration of allantoin, sP-selectin, and FRAP than females (mainly *p* < 0.01).

Blood pressure

Hypertensive participants (defined by a BP above 140/90 mmHg or primary hypertension under therapy) were found to have elevated concentrations of CRP (*p* = 0.03 with placebo) and aldosterone (*p* = 0.01 with febuxostat and rasburicase) than normotensives. Plasma angiotensin II levels were higher in hypertensives than in normotensives; however, the difference was not significant. UA concentrations decreased for the two subgroups with febuxostat and with febuxostat and rasburicase (all *p* < 0.0001). In normotensives participants, the concentration of aldosterone decreased with febuxostat and with febuxostat and rasburicase (ANOVA *p* = 0.02; placebo *vs*. febuxostat *p* = 0.6; placebo *vs*. febuxostat and rasburicase *p* = 0.048 and febuxostat *vs.* febuxostat and rasburicase *p* = 0.07) .

Diastolic and systolic BP and PWV were higher in hypertensive participants. AIx and AIx75 were similar in both subgroups. Induced hypouricemia did not modify hemodynamic parameters in hypertensives. In normotensives, diastolic BP and AIx decreased with febuxostat compared to febuxostat and rasburicase (*p* = 0.009) and placebo (*p* = 0.04), respectively.

Normotensive participants presented better AUC of CVC after acetylcholine iontophoresis with placebo and febuxostat. The late phase AUC of CVC induced by heat after L-NAME iontophoresis was higher in normotensive participants with febuxostat. Heat-induced AUC of CVC after L-NAME was higher febuxostat (*p* <0.05) especially in normotensive participants. No new changes were observed for oxidative stress and other endothelial function markers. However, the reduction in MPO activity was more significant in normotensive than hypertensive participants.

| **Table S1.1. Biological and hemodynamic parameters by sex** | | | | | | | | | | | | | | | | |  |  |
| --- | --- | --- | --- | --- | --- | --- | --- | --- | --- | --- | --- | --- | --- | --- | --- | --- | --- | --- |
|  |  |  |  |  |  |  |  |  |  |  |  |  |  |  |  |  |  |  |
| **Measures** | **Placebo (n = 36)** | |  | **Febuxostat (n = 36)** | |  | **Febuxostat and Rasburicase (n = 36)** | |  | **Females p-value** | | | |  | **Males p-value** | | | |
|  | **Females (13)** | **Males (23)** |  | **Females (13)** | **Males (23)** |  | **Females (13)** | **Males (23)** |  | *Anova* | *P vs. FX* | *P vs. FX-R* | *FX vs.FX-R* |  | *Anova* | *P vs. FX* | *P vs. FX-R* | *FX vs.FX-R* |
|  |  |  |  |  |  |  |  |  |  |  |  |  |  |  |  |  |  |  |
| **Biological** |  |  |  |  |  |  |  |  |  |  |  |  |  |  |  |  |  |  |
| Uric acid (mg/dL)* | 3.8 [3.3 ; 4.7] | 5.8 [5.1 ; 6.2] |  | 1.1 [0.8 ; 1.3] | 2.1 [1.9 ; 2.6] |  | 0.2 [0.2 ; 0.2] | 0.2 [0.2 ; 0.2] |  | **< 0.0001** | **< 0.0001** | **< 0.0001** | **< 0.0001** |  | **< 0.0001 ‡** | **< 0.0001 ‡** | **< 0.0001 ‡** | **< 0.0001 ‡** |
| Urine uric acid/creatinine ratio (mmol/g)* | 49.9 [38.3 ; 55.4] | 29.3 [24.2 ; 41.7] |  | 10.1 [5.7 ; 10.5] | 10.2 [7.8 ; 11.7] |  | 4.6 [2.3 ; 7.4] | 2.5 [1.5 ; 4.6] |  | **< 0.0001 ‡** | **0.003 ‡** | **0.003 ‡** | 0.07 ‡ |  | **<0.0001 ‡** | **< 0.0001 ‡** | **< 0.0001 ‡** | **< 0.0001 ‡** |
|  |  |  |  |  |  |  |  |  |  |  |  |  |  |  |  |  |  |  |
| **Hemodynamic parameters** |  |  |  |  |  |  |  |  |  |  |  |  |  |  |  |  |  |  |
| Systolic BP (mmHg) | 125 (18) | 135 (14) |  | 123 (18) | 132 (17) |  | 125 (16) | 133 (13) |  | 0.5 | - | - | - |  | 0.3 | - | - | - |
| Diastolic BP (mmHg) | 75 (9) | 84 (10) |  | 75 (9) | 82 (10) |  | 76 (8) | 83 (9) |  | 0.5 | - | - | - |  | **0.01** | **0.03** | 1.0 | 0.1 |
| PP (mmHg) | 50 (13) | 51 (9) |  | 48 (10) | 50 (10) |  | 49 (11) | 49 (7) |  | 0,6 | - | - | - |  | 0,6 | - | - | - |
| MAP (mmHg)* | 93 [83 ; 100] | 99 [91 ; 108] |  | 91 [83 ; 100] | 95 [90 ; 103] |  | 93 [85 ; 97] | 99 [94 ; 105] |  | 0,4 | - | - | - |  | **0.008 ‡** | 0.08 ‡ | 0.5 ‡ | 0.2 ‡ |
| HR (/min) | 55 (7) | 58 (7) |  | 59 (7) | 58 (7) |  | 57 (7) | 59 (8) |  | 0.07 | - | - | - |  | 0.8 | - | - | - |
| AIx (%) | 36.6 (7.9) | 26.2 (5.3) |  | 31.9 (10.1) | 25.0 (4.8) |  | 33.5 (7.6) | 25.1 (6.2) |  | **0.001** | **0.004** | **0.05** | 0.3 |  | 0.4 | - | - | - |
| AIx75 (%) | 25.7 (7.2) | 17.6 (5.3) |  | 23.5 (8.3) | 16.6 (5.8) |  | 23.9 (6.9) | 16.7 (6.2) |  | **0.02** | **0.05** | **0.04** | 1.0 |  | 0.5 | - | - | - |
| cr-PWV (m/s) | 6.9 (0.7) | 7.0 (1.0) |  | 6.7 (1.1) | 6.8 (0.5) |  | 6.8 (1.1) | 6.9 (0.5) |  | 0.7 | - | - | - |  | 0.3 | - | - | - |
| cf-PWV (m/s)* | 7.4 [5.8 ; 8.0] | 6.9 [6.1 ; 8.2] |  | 7.2 [6.0 ; 8.1] | 6.7 [6.1 ; 8.5] |  | 7.0 [5.8 ; 7.9] | 7.3 [6.1 ; 8.5] |  | 0.7 | - | - | - |  | 0.1 ‡ | - | - | - |
|  |  |  |  |  |  |  |  |  |  |  |  |  |  |  |  |  |  |  |
| P indicates placebo; FX, febuxostat; FX-R, febuxostat and rasburicase; BP, blood pressure; PP, pulse pressure; MAP, mean arterial pressure ; HR, heart rate; AIx and AIx75: augmentation index and AIx corrected for heart rate; cr- and cf-PWV, carotid-radial and carotid-femoral pulse wave velocity. | | | | | | | | | | | | | | | | | | |
| *: not normally distributed | | | | | | | | | | | | | | | | |  |  |
| ‡ : non parametric p results |  |  |  |  |  |  |  |  |  |  |  |  |  |  |  |  |  |  |

| **Table S1.2. Biological and hemodynamic parameters by hypertension status** | | | | | | | | | | | | | |  |  |  |  |  |
| --- | --- | --- | --- | --- | --- | --- | --- | --- | --- | --- | --- | --- | --- | --- | --- | --- | --- | --- |
|  |  |  |  |  |  |  |  |  |  |  |  |  |  |  |  |  |  |  |
| **Measures** | **Placebo (n = 36)** | |  | **Febuxostat (n = 36)** | |  | **Febuxostat and Rasburicase (n = 36)** | |  | **HTA p-value** | | | |  | **Normotensives p-value** | | | |
|  | **HTA (13)** | **nTA (23)** |  | **HTA (13)** | **nTA (23)** |  | **HTA (13)** | **nTA (23)** |  | *Anova* | *P vs. FX* | *P vs. FX-R* | *FX vs.FX-R* |  | *Anova* | *P vs. FX* | *P vs. FX-R* | *FX vs.FX-R* |
|  |  |  |  |  |  |  |  |  |  |  |  |  |  |  |  |  |  |  |
| **Biological** |  |  |  |  |  |  |  |  |  |  |  |  |  |  |  |  |  |  |
| Uric acid (mg/dL) | 5.4 (1.1) | 4.9 (1.4) |  | 2.2 (1.1) | 1.7 (1.0) |  | 0.3 (0.4) | 0.2 (0.0) |  | **< 0.0001** | **< 0.0001** | **< 0.0001** | **< 0.0001** |  | **< 0.0001** | **< 0.0001** | **< 0.0001** | **< 0.0001** |
| Aldosterone (ng/L)* | 56.1 [51.9 ; 80.6] | 53.3 [35.2 ; 74.1] |  | 62.4 [41.7 ; 76.3] | 48.9 [39.0 ; 64.2] |  | 61.0 [48.2 ; 83.4] | 42.7 [34.2 ; 53.0] |  | 0.9 | - | - | - |  | **0.02 ‡** | 0.6 ‡ | **0.048 ‡** | 0.07 ‡ |
| Urine uric acid/creatinine ratio (mmol/g)* | 29.7 [22.8 ; 47.6] | 38.8 [28.1 ; 51.9] |  | 10.3 [7.4 ; 12.0] | 10.0 [7.0 ; 10.8] |  | 3.1 [1.5 ; 5.1] | 2.8 [1.9 ; 6.3] |  | **< 0.0001 ‡** | **0.003 ‡** | **0.003 ‡** | **0.006 ‡** |  | **< 0.0001 ‡** | **< 0.0001 ‡** | **< 0.0001 ‡** | **< 0.0001 ‡** |
|  |  |  |  |  |  |  |  |  |  |  |  |  |  |  |  |  |  |  |
| **Hemodynamic parameters** |  |  |  |  |  |  |  |  |  |  |  |  |  |  |  |  |  |  |
| Systolic BP (mmHg) | 145 [141 ; 151] | 121 [117 ; 129] |  | 142 [135 ; 150] | 119 [114 ; 128] |  | 140 [135 ; 151] | 125 [118 ; 131] |  | 0.5 ‡ | - | - | - |  | 0.06 | - | - | - |
| Diastolic BP (mmHg) | 89 (9) | 76 (7) |  | 89 (10) | 74 (7) |  | 88 (9) | 76 (6) |  | 0.3 | - | - | - |  | **0.003** | 0.06 | 1.0 | **0.009** |
| PP (mmHg) | 58 (7) | 47 (9) |  | 56 (10) | 48 (8) |  | 55 (6) | 46 (8) |  | 0,5 | - | - | - |  | 0,8 | - | - | - |
| MAP (mmHg)* | 108 [102 ; 113] | 92 [88 ; 97] |  | 103 [99 ; 112] | 90 [84 ; 93] |  | 105 [100 ; 112] | 94 [88 ; 98] |  | 0,6 | - | - | - |  | **0.02 ‡** | 0.07 ‡ | 1.0 ‡ | **0.04 ‡** |
| HR (/min) | 60 (7) | 55 (7) |  | 59 (7) | 58 (7) |  | 60 (9) | 57 (62) |  | 0.9 | - | - | - |  | 0.2 | - | - | - |
| AIx (%) | 28.9 (7.6) | 30.0 (8.2) |  | 27.9 (7.9) | 27.3 (7.7) |  | 27.4 (7.9) | 28.5 (7.7) |  | 0.3 | - | - | - |  | **0.03** | **0.04** | 0.5 | 0.5 |
| AIx75 (%) | 21.1 (7.6) | 20.2 (7.2) |  | 20.3 (7.9) | 18.4 (7.2) |  | 19.3 (6.9) | 19.2 (7.7) |  | 0.1 | - | - | - |  | 0.1 | - | - | - |
| cr-PWV (m/s) | 7.3 (0.7) | 6.8 (1.0) |  | 7.1 (0.7) | 6.5 (1.0) |  | 7.0 (0.7) | 6.8 (1.0) |  | 0.07 | - | - | - |  | 0.1 | - | - | - |
| cf-PWV (m/s)* | 8.2 [7.1 ; 9.5] | 6.6 [5.8 ; 7.4] |  | 8.1 [6.8 ; 9.1] | 6.2 [6.0 ; 7.4] |  | 8.4 [7.3 ; 9.0] | 6.9 [6.0 ; 7.9] |  | 0.2 | - | - | - |  | 0.3 ‡ | - | - | - |
|  |  |  |  |  |  |  |  |  |  |  |  |  |  |  |  |  |  |  |
| P indicates placebo; FX, febuxostat; FX-R, febuxostat and rasburicase; BP, blood pressure; PP, pulse pressure ; MAP, mean arterial pressure ; HR, heart rate; AIx and AIx75: augmentation index and AIx corrected for heart rate; cr- and cf-PWV, carotid-radial and carotid-femoral pulse wave velocity. | | | | | | | | | | | | | | | | | | |
| *: not normally distributed | | | | | | | | | | | | | |  |  |  |  |  |
| ‡ : non parametric p results |  |  |  |  |  |  |  |  |  |  |  |  |  |  |  |  |  |  |

| **Table S2.1. Markers of oxidative stress and endothelial function by sex** | | | | | | | | | | | | | | | | | | | | | |  |  |  |  |
| --- | --- | --- | --- | --- | --- | --- | --- | --- | --- | --- | --- | --- | --- | --- | --- | --- | --- | --- | --- | --- | --- | --- | --- | --- | --- |
|  |  |  |  |  |  |  |  |  |  |  |  |  |  |  |  |  |  |  |  |  |  |  |  |  |  |
| **Measures** |  | **Placebo (n = 36)** | |  | **Febuxostat (n = 36)** | |  | **Febuxostat and Rasburicase (n = 36)** | |  | **Females p-value** | | | | | | |  | **Males p-value** | | | | | | |
|  |  | **Females (13)** | **Males (23)** |  | **Females (13)** | **Males (23)** |  | **Females (13)** | **Males (23)** |  | *Anova* |  | *P vs. FX* |  | *P vs. FX-R* |  | *FX vs.FX-R* |  | *Anova* |  | *P vs. FX* |  | *P vs. FX-R* |  | *FX vs.FX-R* |
|  |  |  |  |  |  |  |  |  |  |  |  |  |  |  |  |  |  |  |  |  |  |  |  |  |  |
| **Oxidative stress** |  |  |  |  |  |  |  |  |  |  |  |  |  |  |  |  |  |  |  |  |  |  |  |  |  |
| Allantoin (µmol/L)* |  | 0.6 [0.5 ; 1.0] | 0.7 [0.5 ; 0.8] |  | 0.4 [0.3 ; 0.4] | 0.6 [0.5 ; 0.6] |  | 14.4 [11.8 ; 15.9] | 24.6 [20.5 ; 33.8] |  | **< 0.0001 ‡** |  | **0.006 ‡** |  | **0.003 ‡** |  | **0.003 ‡** |  | **< 0.0001 ‡** |  | 0.08 ‡ |  | **< 0.0001 ‡** |  | **< 0.0001 ‡** |
| Chloro-Tyrosine/Tyrosine ratio (x 10^-5^)* |  | 6.5 [5.9 ; 6.8] | 5.5 [4.9 ; 6.9] |  | 6.8 [6.0 ; 8.1] | 6.2 [4.6 ; 6.9] |  | 6.8 [6.0 ; 7.2] | 6.4 [4.8 ; 8.0] |  | 0.2 ‡ |  | **-** |  | **-** |  | **-** |  | 0,08 |  | **-** |  | **-** |  | **-** |
| Homocitrulline/Lysine ratio (x 10^-5^)* |  | 118.4 [104.8 ; 138.8] | 110.2 [93.3 ; 125.5] |  | 114.5 [99.7 ; 141.3] | 115.1 [100.0 ; 129.9] |  | 114.8 [95.3 ; 145.0] | 120.9 [99.0 ; 132.5] |  | 0.8 ‡ |  | **-** |  | **-** |  | **-** |  | 0,1 |  | **-** |  | **-** |  | **-** |
| MPO activity (mU/mL) |  | 0.9 [0.5 ; 1.1] | 1.0 [0.9 ; 1.6] |  | 0.8 [0.4 ; 1.2] | 1.0 [0.6 ; 1.3] |  | 0.4 [0.1 ; 0.5] | 0.2 [0.1 ; 0.4] |  | **0.009 ‡** |  | 1.0 ‡ |  | **0.02 ‡** |  | **0.03 ‡** |  | **< 0.0001 ‡** |  | 0.9 ‡ |  | **< 0.0001 ‡** |  | **< 0.0001 ‡** |
| MPO (ng/mL)*,† |  | 131.2 [106.2 ; 172.7] | 115.6 [79.4 ; 196.7] |  | 149.6 [121.9 ; 175.0] | 126.3 [71.6 ; 176.0] |  | 152.0 [81.9 ; 200.2] | 141.6 [88.3 ; 210.1] |  | 0.6 ‡ |  | **-** |  | **-** |  | **-** |  | 0.6 ‡ |  | **-** |  | **-** |  | **-** |
| MDA (nmol/mL)* |  | 1.4 [1.2 ; 5.4] | 1.3 [1.2 ; 1.6] |  | 1.4 [1.1 ; 5.1] | 1.3 [1.2 ; 1.8] |  | 1.3 [1.1 ; 4.8] | 1.3 [1.3 ; 1.7] |  | 0.3 ‡ |  | **-** |  | **-** |  | **-** |  | 0.2 ‡ |  | **-** |  | **-** |  | **-** |
| FRAP (µmol/L) |  | 1118.6 (172.7) | 1458.2 (226.4) |  | 704.7 (102.3) | 911.0 (142.9) |  | 549.1 (69.9) | 619.5 (77.2) |  | **< 0.0001** |  | **< 0.0001** |  | **< 0.0001** |  | **< 0.0001** |  | **< 0.0001** |  | **< 0.0001** |  | **< 0.0001** |  | **< 0.0001** |
| SOD activity (mU/mL)*^,†^ |  | 0.6 [0.5 ; 0.7] | 0.6 [0.5 ; 0.6] |  | 0.7 [0.6 ; 0.8] | 0.7 [0.6 ; 0.7] |  | 0.7 [0.7 ; 0.8] | 0.7 [0.6 ; 0.7] |  | **< 0.0001** |  | **< 0.0001** |  | **< 0.0001** |  | 1,0 |  | **< 0.0001 ‡** |  | **< 0.0001 ‡** |  | **0.003 ‡** |  | 1.0 ‡ |
|  |  |  |  |  |  |  |  |  |  |  |  |  |  |  |  |  |  |  |  |  |  |  |  |  |  |
| **Endothelial function** |  |  |  |  |  |  |  |  |  |  |  |  |  |  |  |  |  |  |  |  |  |  |  |  |  |
| L-NAME late phase AUC of CVC |  | 29.5 (7.0) | 25.6 (7.3) |  | 32.9 (7.8) | 28.9 (5.7) |  | 30.7 (6.6) | 27.9 (5.3) |  | 0.1 |  | - |  | - |  | - |  | **0.02** |  | **0.04** |  | 0.2 |  | 0.9 |
| S late phase AUC of CVC |  | 33.2 (7.3) | 29.8 (6.7) |  | 36.1 (9.8) | 31.0 (7.1) |  | 34.5 (8.6) | 30.8 (6.0) |  | **0.04** |  | **0.045** |  | 0.7 |  | 0.7 |  | 0.5 |  | - |  | - |  | - |
|  |  |  |  |  |  |  |  |  |  |  |  |  |  |  |  |  |  |  |  |  |  |  |  |  |  |
| P indicates placebo ; FX, febuxostat ; FX-R, febuxostat and rasburicase ; MPO, myeloperoxidase ; MDA, malondialdehyde ; FRAP, Ferric reducing antioxidant power; SOD, superoxide dismutase; L-NAME, L-NG-Nitro arginine methyl ester; and S, saline. | | | | | | | | | | | | | | | | | | | | | | | | | |
| *: not normally distributed | | | | | | | | | | | | | | | | | | | | | |  |  |  |  |
| †: n = 22 for males |  |  |  |  |  |  |  |  |  |  |  |  |  |  |  |  |  |  |  |  |  |  |  |  |  |
| ‡ : non parametric p results |  |  |  |  |  |  |  |  |  |  |  |  |  |  |  |  |  |  |  |  |  |  |  |  |  |

| **Table S2.2. Markers of oxidative stress and endothelial function by hypertension status** | | | | | | | | | | | | | | | | | | | | | |  |  |  |  |
| --- | --- | --- | --- | --- | --- | --- | --- | --- | --- | --- | --- | --- | --- | --- | --- | --- | --- | --- | --- | --- | --- | --- | --- | --- | --- |
|  |  |  |  |  |  |  |  |  |  |  |  |  |  |  |  |  |  |  |  |  |  |  |  |  |  |
| **Measures** |  | **Placebo (n = 36)** | |  | **Febuxostat (n = 36)** | |  | **Febuxostat and Rasburicase (n = 36)** | |  | **HTA p-value** | | | | | | |  | **Normotensives p-value** | | | | | | |
|  |  | **HTA (13)** | **nTA (23)** |  | **HTA (13)** | **nTA (23)** |  | **HTA (13)** | **nTA (23)** |  | *Anova* |  | *P vs. FX* |  | *P vs. FX-R* |  | *FX vs.FX-R* |  | *Anova* |  | *P vs. FX* |  | *P vs. FX-R* |  | *FX vs.FX-R* |
|  |  |  |  |  |  |  |  |  |  |  |  |  |  |  |  |  |  |  |  |  |  |  |  |  |  |
| **Oxidative stress** |  |  |  |  |  |  |  |  |  |  |  |  |  |  |  |  |  |  |  |  |  |  |  |  |  |
| Allantoin (µmol/L)* |  | 0.7 [0.5 ; 0.9] | 0.6 [0.5 ; 0.8] |  | 0.6 [0.4 ; 0.6] | 0.5 [0.4 ; 0.6] |  | 24.6 [13.5 ; 39.9] | 18.6 [12.7 ; 24.1] |  | **< 0.0001 ‡** |  | **0.03 ‡** |  | **0.003 ‡** |  | **0.003 ‡** |  | **<0.0001 ‡** |  | **0.009 ‡** |  | **< 0.0001 ‡** |  | **< 0.0001 ‡** |
| Chloro-Tyrosine/Tyrosine ratio (x 10^-5^)* |  | 5.5 [4.9 ; 6.9] | 6.2 [5.4 ; 6.9] |  | 6.6 [5.0 ; 7.2] | 6.3 [5.5 ; 6.9] |  | 6.6 [4.8 ; 8.2] | 6.7 [5.6 ; 7.3] |  | 0,3 |  | **-** |  | **-** |  | **-** |  | 0.8 ‡ |  | **-** |  | **-** |  | **-** |
| Homocitrulline/Lysine ratio (x 10^-5^)* |  | 110.2 [98.7 ; 127.1] | 114.0 [95.9 ; 128.6] |  | 115.1 [102.9 ; 140.0] | 108.9 [96.3 ; 131.3] |  | 120.9 [101.9 ; 142.2] | 112.8 [95.3 ; 132.5] |  | 0,5 |  | **-** |  | **-** |  | **-** |  | 0.7 ‡ |  | **-** |  | **-** |  | **-** |
| MPO activity (mU/mL) |  | 1.0 [0.8 ; 1.2] | 1.0 [0.8 ; 1.4] |  | 0.8 [0.7 ; 1.2] | 1.0 [0.6 ; 1.3] |  | 0.2 [0.1 ; 0.7] | 0.2 [0.1 ; 0.4] |  | **0.05 ‡** |  | 1.0 ‡ |  | 0.07 ‡ |  | 0.1 ‡ |  | **<0.0001 ‡** |  | 1.0 ‡ |  | **< 0.0001 ‡** |  | **< 0.0001 ‡** |
| MPO (ng/mL)*,† |  | 92.3 [63.2 ; 176.8] | 134.3 [109.4 ; 181.3] |  | 83.5 [56.0 ; 126.3] | 157.3 [135.8 ; 209.6] |  | 124.8 [66.2 ; 193.1] | 155.5 [96.2 ; 205.9] |  | 0.1 ‡ |  | **-** |  | **-** |  | **-** |  | 0.06 ‡ |  | **-** |  | **-** |  | **-** |
| MDA (nmol/mL)* |  | 1.4 [1.2 ; 6.6] | 1.4 [1.2 ; 1.6] |  | 1.4 [1.2 ; 5.6] | 1.3 [1.2 ; 1.8] |  | 1.3 [1.2 ; 6.0] | 1.3 [1.2 ; 1.7] |  | 0.8 ‡ |  | **-** |  | **-** |  | **-** |  | 0.1 ‡ |  | **-** |  | **-** |  | **-** |
| FRAP (µmol/L) |  | 1438.7 (252.4) | 1277.3 (257.5) |  | 892.3 (195.1) | 805.0 (136.2) |  | 637.2 (88.3) | 569.8 (67.1) |  | **< 0.0001** |  | **< 0.0001** |  | **< 0.0001** |  | **< 0.0001** |  | **<0.0001** |  | **<0.0001** |  | **<0.0001** |  | **<0.0001** |
| SOD activity (mU/mL)*^,†^ |  | 0.6 [0.5 ; 0.7] | 0.5 [0.5 ; 0.6] |  | 0.7 [0.6 ; 0.8] | 0.7 [0.6 ; 0.7] |  | 0.7 [0.6 ; 0.8] | 0.7 [0.6 ; 0.7] |  | **0.03 ‡** |  | **0.009 ‡** |  | **0.048 ‡** |  | 1,0 |  | **<0.0001** |  | **<0.0001** |  | **<0.0001** |  | 1,0 |
|  |  |  |  |  |  |  |  |  |  |  |  |  |  |  |  |  |  |  |  |  |  |  |  |  |  |
| **Endothelial function** |  |  |  |  |  |  |  |  |  |  |  |  |  |  |  |  |  |  |  |  |  |  |  |  |  |
| ACh full AUC of CVC |  | 33.2 (5.0) | 39.4 (7.9) |  | 33.3 (7.5) | 38.7 (7.3) |  | 33.7 (6.9) | 39.0 (8.6) |  | 0.9 |  | - |  | - |  | - |  | 0.8 |  | - |  | - |  | - |
| SNP full AUC of CVC |  | 29.7 (5.6) | 32.7 (7.1) |  | 29.5 (8.2) | 34.5 (7.4) |  | 30.2 (5.4) | 33.4 (9.2) |  | 1.0 |  | - |  | - |  | - |  | 0.4 |  | - |  | - |  | - |
| L-NAME late phase AUC of CVC |  | 26.3 (3.4) | 27.4 (8.8) |  | 27.3 (4.1) | 32.1 (7.3) |  | 28.8 (4.5) | 29.0 (6.6) |  | **0.02** |  | 0.5 |  | 0.09 |  | 0.2 |  | **0.009** |  | **0.03** |  | 1.0 |  | **0.047** |
| S late phase AUC of CVC |  | 28.1 (5.8) | 32.7 (7.2) |  | 29.8 (7.2) | 34.6 (8.7) |  | 29.8 (6.3) | 33.4 (7.5) |  | 0.1 |  | - |  | - |  | - |  | 0.2 |  | - |  | - |  | - |
|  |  |  |  |  |  |  |  |  |  |  |  |  |  |  |  |  |  |  |  |  |  |  |  |  |  |
| P indicates placebo ; FX, febuxostat ; FX-R, febuxostat and rasburicase ; MPO, myeloperoxidase ; MDA, malondialdehyde ; FRAP, Ferric reducing antioxidant power; SOD, superoxide dismutase; ACh, acetylcholine; SNP, sodium nitroprusside; L-NAME, L-NG-Nitro arginine methyl ester; and S, saline. | | | | | | | | | | | | | | | | | | | | | | | | | |
| *: not normally distributed | | | | | | | | | | | | | | | | | |  |  |  |  |  |  |  |  |
| †: n = 22 for normotensives | | | | | |  |  |  |  |  |  |  |  |  |  |  |  |  |  |  |  |  |  |  |  |
| ‡ : non parametric p results |  |  |  |  |  |  |  |  |  |  |  |  |  |  |  |  |  |  |  |  |  |  |  |  |  |

1. **Supplemental references**

1 Delporte C, Franck T, Noyon C, Dufour D, Rousseau A, Madhoun P, *et al.* Simultaneous measurement of protein-bound 3-chlorotyrosine and homocitrulline by LC-MS/MS after hydrolysis assisted by microwave: Application to the study of myeloperoxidase activity during hemodialysis. Talanta 2012; **99**:603–609.

2 Turner R, Stamp LK, Kettle AJ. Detection of allantoin in clinical samples using hydrophilic liquid chromatography with stable isotope dilution negative ion tandem mass spectrometry. J Chromatogr B 2012; **891**–**892**:85–89.

3 Franck T, Kohnen S, Zouaoui-Boudjeltia K, Van Antwerpen P, Bosseloir A, Niesten A, *et al.* A new easy method for specific measurement of active myeloperoxidase in human biological fluids and tissue extracts. Talanta 2009; **80**:723–729.

4 Wauters A, Dreyfuss C, Pochet S, Hendrick P, Berkenboom G, van de Borne P, *et al.* Acute exposure to diesel exhaust impairs nitric oxide-mediated endothelial vasomotor function by increasing endothelial oxidative stress. Hypertension 2013; **62**:352–358.
